# An atypical imaging characteristic of calvarial metastasis of neuroblastoma as multiple multi-loculated cystic masses with internal blood-fluid levels: a case report

**DOI:** 10.1186/s12883-022-03001-9

**Published:** 2022-12-09

**Authors:** Samin Khoei, Mohammad Reza Boustani, Neda Pak

**Affiliations:** 1 Radiology Department, Imam Khomeini Hospital Complex, Tehran University of Medical Sciences, Tehran, Iran; 2 Department of Neurosurgery, AJA University of Medical Sciences, Tehran, Iran; 3 Department of Neurosurgery, Dr. Shariati Hospital, Tehran University of Medical Sciences, Tehran, Iran; 4 Department of Radiology, Children’s Medical Center of Excellence and Dr. Shariati Hospital, Tehran University of Medical Sciences, Tehran, Iran

**Keywords:** Neuroblastoma, Metastasis, MRI, Epidural, Case report

## Abstract

**Background:**

As the third most common malignancy of childhood, Neuroblastoma has a great propensity to metastasize to multiple organs. The most common site of metastasis is the bone and bone marrow. Concerning the central nervous system, neuroblastoma usually involves the calvarium and the external dural surface. The skull metastases may show different appearances, including: multiple lytic bone lesions, bone thickening, hair-on-end periosteal reaction, irregular suture widening and/or plaque like epidural deposits. Here we present a case of metastatic neuroblastoma, appearing as multiple multi-loculated cystic epidural masses with internal blood-fluid levels as a rare imaging manifestation of calvarial metastasis.

**Case presentation:**

An 8-year-old boy with known history of autism, presented to the emergency department with a 3-month history of intermittent fever, malaise and myalgia and headache along with significant weight loss. Laboratory examination revealed elevated ESR and CRP and anemia. On Abdomino-Pelvic imaging a well-defined, 45*30*24 mm, solid-cystic mass was observed, replacing the normal left adrenal gland. On brain MRI, multiple multi-loculated cystic, lentiform masses were observed on the external surface of cerebral hemispheric dura. Multiple fluid–fluid levels were noted in the locules in some of which the dependent fluid was hyperintense on T1w and FLAIR and hypointense on T2w sequences, compatible with blood, representing blood-fluid level. The wall and septa of the masses, enhanced after contrast administration. Associated abnormal marrow signal and aggressive type periosteal reactions were identified in the overlying bone. All of the lesions had increased uptake in MIBG scan. Bone marrow biopsy revealed small round cells, diagnostic for neuroblastoma. The patient underwent chemotherapy treatment. All calvarial/epidural metastatic lesions resolved after chemotherapy and residual adrenal tumor was resected.

**Conclusion:**

Cystic epidural lesions, especially when associated with adjacent abnormal bone marrow signal, or periosteal reaction and containing blood-fluid level should raise the suspicion of a calvarial metastasis.

## Background

As the third most common malignancy of childhood, Neuroblastoma has a great propensity to metastasize to multiple organs. The primary tumor originates from the adrenal gland or sympathetic ganglia. The most common site of metastasis is the bone and bone marrow. Concerning the central nervous system, neuroblastoma usually involves the calvarium and the external dural surface, which acts as a barrier against direct invasion to leptomeninges; thus metastasis to brain parenchyma and leptomeninges are rare [1–3]. The primary mass is usually solid and shows variable contrast enhancement, eventual foci of necrosis, calcification and hemorrhage [1]. The skull metastases may show different appearances, including: multiple lytic bone lesions, bone thickening, hair-on-end periosteal reaction, irregular suture widening and/or plaque like epidural deposits [1, 4].

## Case presentation

**Fig. 1:**
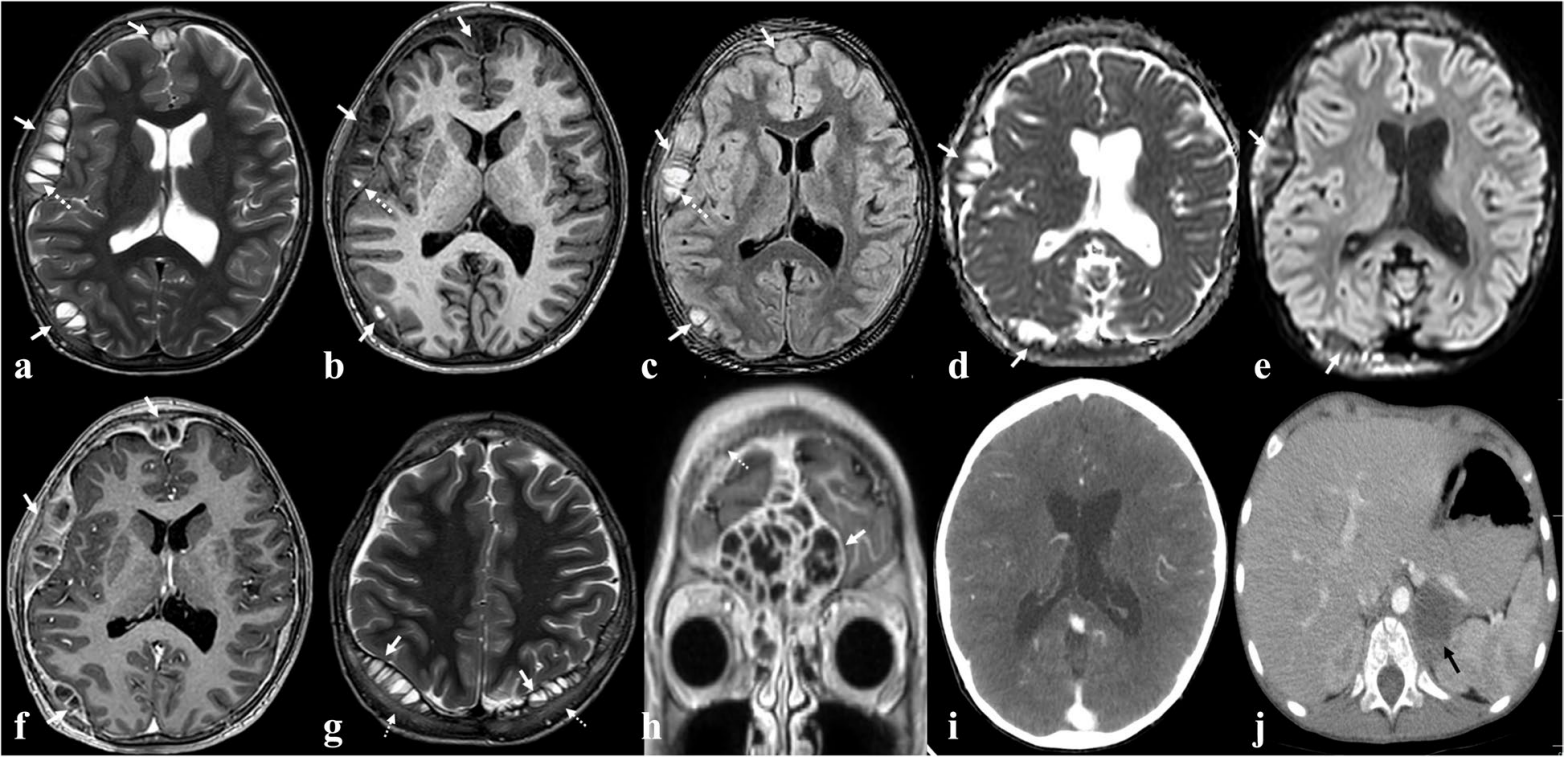
Brain MRI with and without contrast media administration [**a** to **h**] revealed multifocal multi-loculated cystic lesions in the epidural space [white arrows in all images] containing fluid–fluid levels [dashed white arrows in **a**, **b** and **c**] which the dependent fluid was hyperintense on axial T1w and FLAIR [**b** and **c** respectively] and hypointense on axial T2w [A] sequences compatible with blood representing blood-fluid level. Diffuse pachymeningeal enhancement and enhancement of wall and septa of lesions are noted on axial contrast-enhanced T1w sequence [**f**]. Image [**g**] shows involvement of overlying calvarial bones as hyperintense signal change on axial T2 w sequence [dashed white arrows]. DWI and ADC map sequences [**e** and **d**] show no restriction. Image [**h**] is coronal contrast-enhanced T1w sequences showing similar large epidural metastatic lesion around flax [white arrow], note the calvarial involvement of frontal bone as abnormal bone and adjacent dural enhancement with overlying enhancing soft tissue component [dashed white arrow]. Follow up CT scan after chemotherapy treatment [**i**] shows complete resolution of meningeal lesions. Image **j** shows low attenuating soft tissue mass in left adrenal gland [black arrow]

**Fig. 2:**
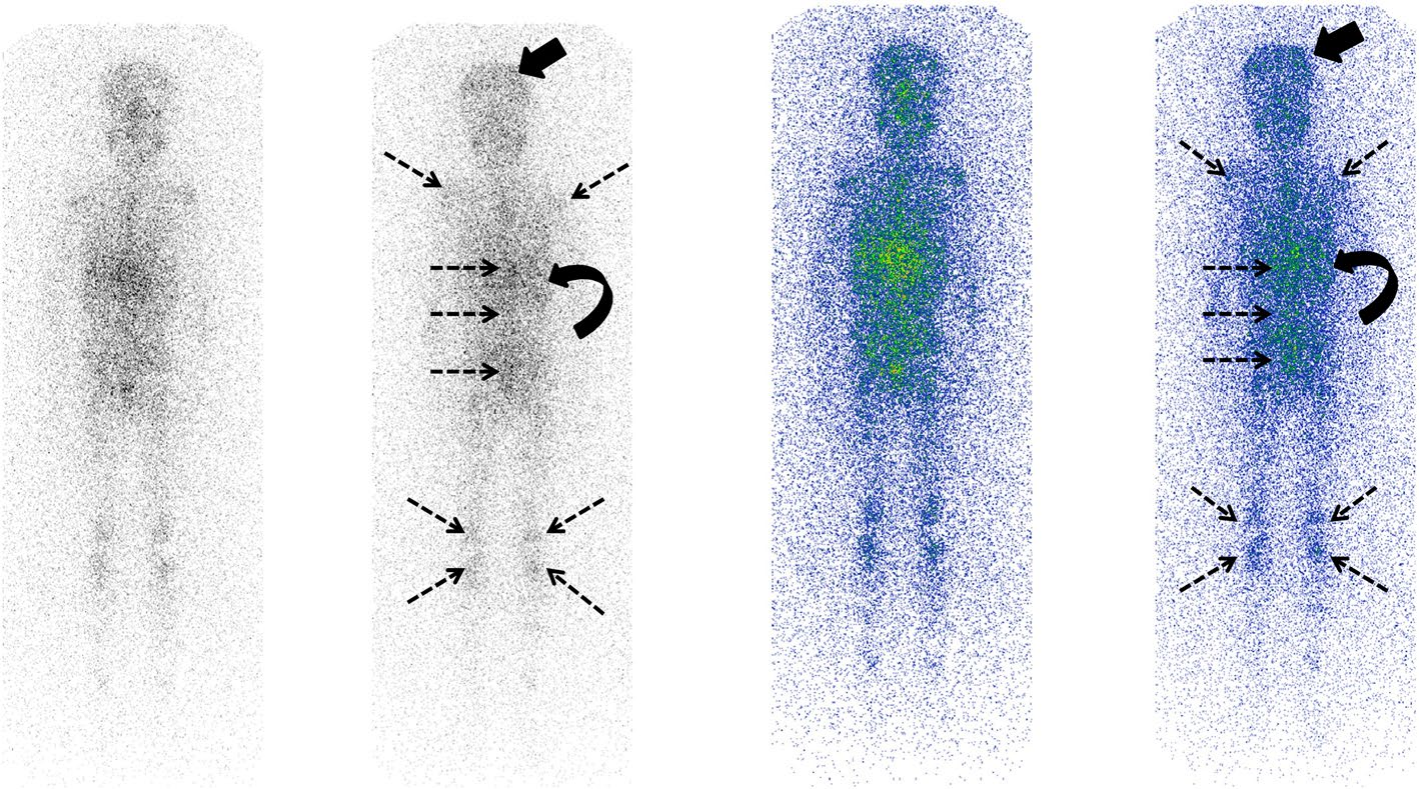
The initial MIBG scintigraphy of the patient performed after IV injection of 1 mCi 131I-MIBG confirms avid uptake of radiotracer in the calvarial lesions [solid black arrow], along with diffuse skeletal [dashed black arrows] and left adrenal mass [curved black arrow] uptake

**Fig. 3:**
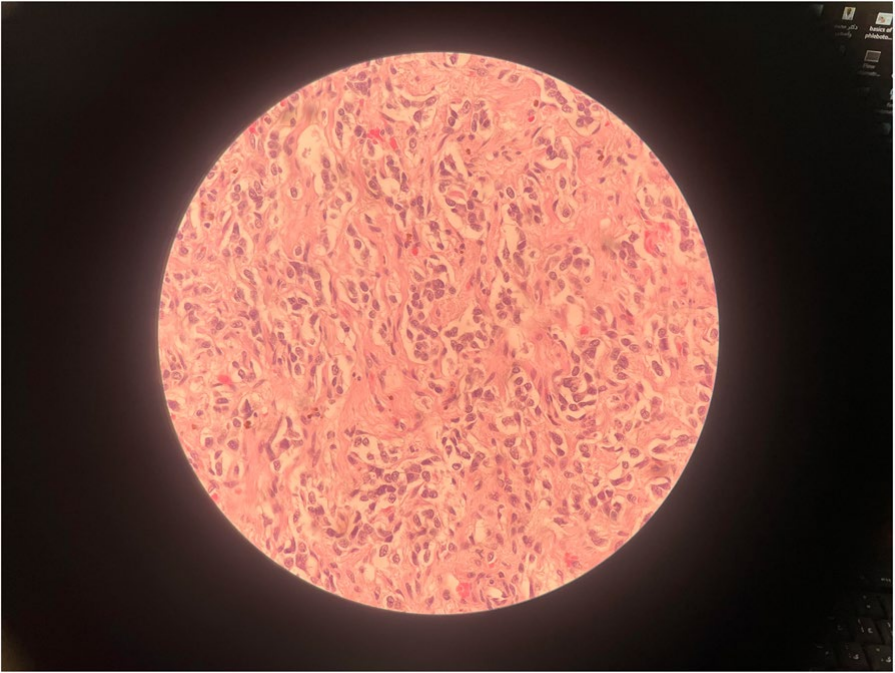
Microscopic tissue exam of the left adrenal mass, undergone Hematoxylin and Eosin staining, with 400 × magnification, shows small round blue cells confirming the diagnosis of neuroblastoma

An 8-year-old boy with known history of autism, presented to the emergency department with a 3-month history of intermittent fever, malaise and myalgia and headache along with significant weight loss. Other than autism, no past medical history existed. His drug history included Imipramine and Haloperidol. Except for fever [38 degrees centigrade], pale conjunctivae and palpable soft bulging in forehead, physical examination was unremarkable. Laboratory examination revealed ESR: 125 mm/hr, CRP: 90 mg/L, Hb: 7.1 g/dL, LDH: 1789U/L, ferritin: 2570 µg/L and TSH: 7.77mIU/L. On Abdomino-Pelvic ultrasound, a well-defined, 45*30*24 mm, solid-cystic mass was observed, replacing the normal left adrenal gland, with multiple pathologic lymph nodes in the para-aortic chain. Thoraco-Abdomino-Pelvic CT scan (Fig. 1), revealed the heterogeneous enhancing left adrenal mass with central necrosis and multiple pathologic left para-aortic lymphadenopathies. The heterogeneous bone marrow density of the vertebral bodies, lytic lesions in the right scapula and 4^th^ right rib suggested bony metastases. On brain MRI, multiple multi-loculated cystic, lentiform masses were observed on the external surface of cerebral hemispheric dura. Multiple fluid–fluid levels were noted in the locules in some of which, the dependent fluid was hyperintense on T1w and FLAIR and hypointense on T2w sequences, compatible with blood, representing blood-fluid level. The wall and septa of the masses, enhanced after contrast administration. Associated abnormal marrow signal and aggressive type periosteal reaction were identified in the overlying bone (Fig. 1). MIBG scan showed increased uptake, compatible with the calvarial/epidural lesions, all vertebrae, bilateral humeri, ribs and pelvic bones, femurs and tibiae and in the left adrenal mass (Fig. 2). Bone marrow biopsy revealed small round cells, diagnostic for neuroblastoma. The patient underwent chemotherapy treatment. All calvarial/epidural metastatic lesions resolved after chemotherapy and the residual adrenal tumor was resected. The biopsy specimen of the resected adrenal mass also confirmed the diagnosis of neuroblastoma (Fig. 3). Further chemotherapy was done, and no primary or metastatic recurrence was observed in the first year of post treatment follow-up.

## Discussion

As the most common extracranial solid tumor of childhood, neuroblastoma accounts for 10% of childhood malignancies, and 15% of malignancy-related deaths [3, 5, 6]. In the majority of cases the disease manifests before 4 years of age [6]. Metastases are present in 70% of patients at the time of diagnosis, the most frequent site of which is bone [3]. Neuroblastoma is derived from the neural crest cells, aimed to develop into the sympathetic system. Owing to the wide range of neuronal differentiation of these original cells, this tumor shows a wide range of manifestations, thus the name “The great imitator” [3, 5] new chemotherapeutic regimens have improved the survival rate of the patients, which makes it more probable for us to face with new appearances of neuroblastoma metastases, especially in the CNS – as a sanctuary for tumor cells- [5]. Calvarial/dural metastases occur in 25% of patients, and may sometimes precede the diagnosis of the main primary tumor [3, 6, 7]. As this hypothesis has been recently mentioned, that calvarial/dural metastases may be a potential origin of the brain parenchymal disease spread, identification of this entity becomes more critical in the early stage, to prevent further dissemination [8]. Thus, as a radiologist, we should be familiar with typical and atypical imaging appearances of calvarial/dural metastases of neuroblastoma.

The skull metastases may show different appearances, including: multiple lytic bone lesions, bone thickening, hair-on-end periosteal reaction, irregular suture widening and/or plaque like epidural deposits [3, 4]. Dural metastases usually appear as increased enhancement, thickening or plaque, which are most commonly located at the extradural surface [3]. To our knowledge multi-loculated cystic epidural lesions have not been reported previously as an imaging feature of calvarial/epidural metastatsis of neuroblastoma.

Necrosis and hemorrhage have been mentioned as the secondary histologic features of neuroblastoma [1]. Although rarely occurring; cystic degeneration, accompanied by hemorrhagic components, has been mentioned in the brain parenchymal metastases of neuroblastoma. The underlying pathogenesis of cyst formation in parenchymal brain masses, has said to be associated with hemorrhagic necrosis, or disruption of the blood–brain-barrier with the resultant accumulation of plasma exudate [2].

Our patient, who first presented with non-specific symptoms, and last was confirmed to be a case of neuroblastoma, showed an atypical imaging appearance of calvarial metastasis: multiple multi-loculated cystic masses, with enhancing wall and septa, which contain blood-fluid levels, and were located on the external dural surface. No previous case of calvarial/epidural metastasis of neuroblastoma with cystic-hemorrhagic degeneration has been reported in the literature, which may cause unfamiliarity of the radiologists with this appearance of the entity, thus leading to incorrect interpretations [1, 3, 5]. Associated abnormal marrow signal and aggressive periosteal reaction in the overlying bone, are clues to correct diagnosis. The diagnosis was confirmed on MIBG scan and bone marrow biopsy.

## Conclusion

Cystic-hemorrhagic epidural lesions, especially when associated with adjacent abnormal bone marrow signal or periosteal reaction should raise the suspicion of a calvarial/epidural metastasis. Neuroblastoma should be kept in mind in children as the most common primary of these metastatic lesions, in the appropriate clinical setting.

## Data Availability

The datasets used during the current study are available from the corresponding author on reasonable request.

## Abbreviations

ESR: Erythrocyte sedimentation rate
CRP: C-reactive protein
Hb: Hemoglobin
LDH: Lactate dehydrogenase
TSH: Thyroid stimulating hormone
CT scan: Computerized tomography
MRI: Magnetic resonance imaging
FLAIR: Fluid-attenuated inversion recovery
MIBG: Meta-iodobenzylguanidine
CNS: Central nervous system
